# The key role of vasa vasorum inherited remodeling in QBS microcirculatory theory of atherosclerosis

**DOI:** 10.3389/fgene.2013.00055

**Published:** 2013-04-16

**Authors:** Sergio Stagnaro, Simone Caramel

**Affiliations:** Department of Advanced Research, International Society of Quantum Biophysical Semeiotics, Board of directors, Research CenterTreviso, Italy

## Introduction

In the present article, the Authors spread their clinical understanding of genetics and biology of heart failure directly from the bedside investigation of biological systems dynamics through an original diagnostic method with the simple use of the stethoscope, i.e., assessing the splenic arrhythmia (Stagnaro, 1985a) or Morosini's syndrome (Stagnaro and Caramel, 2012a).

Cardiovascular disease (CVD), today's growing epidemic, is the first cause of death in world-wide. Despite all the current researches, there are still many open questions, such as the well-localized origin of initial endothelial dysfunction and the relationship between genetic causes and environmental risk factors (about 300!) in atherogenesis. For example, not all patients with intense hyper-dyslipidemia are atherosclerotic, and there are individuals suffering of acute myocardial infarction (AMI), in spite of absent dyslipidemia and type 2 DM.

On the other hand, one wonders why a large number of patients with hyperinsulinemia-insulin-resistance, hypertension, hyper dyslipidemia live to old age without getting CVD. On the contrary, even without environmental risk factors for atherosclerosis (ATS), in several patients we can see the onset of AMI and stroke. Notoriously, CVD occurs even in individuals without common risk factors, while the CVD may be absent in smoking subjects with impaired blood counts and hypertension.

## The pathogenesis of atherosclerosis

The pathogenesis of ATS in the first decade of life and the reasons why the ATS arises in well-localized area and evolves differently, both in the single patient, from artery to artery and from patient to patient, are till now unknown and not yet clinically explored.

We propose that a subject clinically health could be at inherited real risk (IRR) of ATS (Stagnaro, 2009a) or involved by ATS in its different pre-clinical stages (Stagnaro and Caramel, 2012b), although silent, asymptomatic, even if usual clinical tests are normal and they do not reveal any abnormality. According to the studies of one of the authors, this is due to genetic alteration of mit-DNA, evidenced by the “Congenital Acidosic Enzyme-Metabolic Histangiopathy” (CAEMH), a unique functional mitochondrial cytopathy that is present at birth and open a new, original way to medical diagnosis and therapy (Stagnaro, 1985). This is possible with the aid of “Quantum Biophysics Semeiotics” (QBS) (Stagnaro and Stagnaro-Neri, 2004a), a new discipline in the medical field and an extension of the classical semeiotics with a scientific trans-disciplinary approach, i.e., with the support of quantum and complexity theories and its genetics and epigenetics applications (Stagnaro-Neri and Stagnaro, 1997; Stagnaro and Caramel, 2011a).

CAEMH is the *conditio sine qua non* of inherited degenerative pathologies such as cancer (Stagnaro and Caramel, 2013a), T2DM (Stagnaro and Caramel, 2013b), cerebral disorders (Marchionni et al., 2013), and CVD (Stagnaro and Caramel, 2013c). The presence of intense CAEMH in a well-defined area (i.e., heart coronary artery, e.g., of atria, in case of Arrhythmic Constitution) is due to gene mutations in both n-DNA and mit-DNA (Caramel and Stagnaro, 2010). This is the basis for one or more QBS constitutions (Stagnaro and Stagnaro-Neri, 2004b), e.g., atherosclerotic QBS constitution, which could bring about their respective IRRs, i.e., IRR of CVD characterized by microcirculatory remodeling, intense under environmental risk factors.

According to Angiobiopathy theory (Stagnaro, 2009b) microvessels, related parenchyma and genome (i.e., mit-DNA and n-DNA) are intimately related, so that the study of microvascular oscillations can give us valuable information on related parenchyma's patho-physiology (Stagnaro, 2013).

CAEMH modifies mit-DNA of parenchyma as well as vessel wall cells, including those of Vasa Vasorum. Parenchymal alterations parallel the related cell alterations of vessel wall. Parenchymal cells need of less blood supply than the normal one, bringing about vasa vasorum microcirculatory remodeling. In this case, we can clinically observe, from a functional QBS point of view, an impairment between vasomotility and vasomotion of the related microvessels' oscillations, which reflects structural new-born abnormalities represented by type I, subtype (b) aspecific, new-born pathological endoarteriolar blocking devices (EBDs) (Stagnaro, 2009c). As a consequence, arteriovenous anastomoses (AVA) are persistently open and there is the so-called centralization of blood-flow, which causes endothelial damage, brought about by the venous hypertension in nutritional capillaries, tissue acidosis, thus SMC proliferation (polyamines augmentation), and finally SMC migration (FMFs). As a matter of facts, the IRR of CVD is associated to heritable endothelial dysfunction, bed-side recognizable in an easy and reliable way, even from birth, at rest as well as under stress tests (Stagnaro, 2008).

## The QBS microcirculatory theory of atheroscerosis

We suggest a quantum biophysical semeiotics microcirculatory theory of ATS, based on heritable microcirculatory events observed with the stethoscope in every arterial wall, from the birth and during the first decade of life. It provides original and satisfactory solutions to problems so far open.

From the moment of birth, this theory allows us to recognize, by means of Antognetti's Sign, and locate, with the aid of Caotino's Sign (Stagnaro, 2012), on a very large scale and without any monetary cost, the IRR of CVD, as well as the predisposition to arterial calcification (Stagnaro, 1977; Stagnaro and Caramel, 2012c).

According to our theory, the process of atherogenesis starts from birth in individuals suffering from Arteriosclerotic Constitution-Dependent IRR of CVD, even when there is generally no blood dyscrasia of any kind, and blood pressure is normal. The first, initial, fundamental stage of the Natural History of the ATS is the CAEMH.

To summarize, the proposed “QBS microcirculatory theory of atherosclerosis” (Stagnaro and Caramel, 2012d) is enlightening the following paramount aspects: heritable endothelial impairment, caused by intense localized CAEMH and worsened, though not necessarily, by a lot of environmental risk factors, only partially known, brings about lowering synthesis of e-NO radicals, increased secretion of vasoconstrictors substances, so that the endothelial-dependent hemostatic unbalance can predispose, in the examined individuals, to blood mononuclear cells, e.g., monocytes, NT-cell, and platelets, adhesion, due to the increased endothelial synthesis of VCAM-1, ICAM-1, a.s.o., and medial smooth muscle cells proliferation. Their migration to the intimae, monocytes-derived macrophages, as well as lipoproteins storage in the arterial wall, will be observed with the progression of the pre-metabolic syndrome toward the metabolic one.

As briefly mentioned above, we highlight three key points at the base of our theory, that give satisfactory answers to till now un-resolved problems, concerning the physio-pathology of atherogenesis:

1. The presence of a mitochondrial cytopathy, mainly functional, termed CAEMH;
2. the phenomenon of mitochondrial heteroplasmy, both intra and extra-cellular;
3. the well-circumscribed acidosis in the arterial wall, initiating in the second year of life, caused by the remodeling of the vasa vasorum, and the subsequent permanent hyperstomy of AVA, as well as hypertension in nutrition capillary at the precise site of vasa vasorum remodeling, dependent of genome alteration of local parenchymal cells, most affected by CAEMH.

The clinical QBS diagnosis in the first decade of life, starting from the birth, and the analysis of the collected information provide a compelling explanation for the onset of vessel-wall acidosis, caused by the remodeling of the vasa vasorum. It explains thus the phenomenon and important role of mitochondrial heteroplasmy and the circumscribed endothelial dysfunction (HP Zone endothels) (Stagnaro, 2011; Stagnaro and Caramel, 2011b).

On the basis of these data a clinical satisfactory explanation of the penetration—in pathological quantities—in the sub-endothelial space of molecules rich in lipids is possible, even in the absence of significant dyslipidemia—almost never present in these initial stages—and their retention, followed by immobilization in the matrix of the intimae by a physiological acid sphingomyelinase, that only in such an environment, by reduced pH, can carry out its activities in an optimal manner.

## Inherited real risk of cardiovascular disease: bedside diagnosis

The objective QBS examination allows physician to bedside recognize and quantify, in a few minutes, the presence of “IRR” of ATS or overt ATS, even initial, the precise site, its severity, and its evolution monitored over time, through the evaluation of several semeiotics signs, i.e., assessing vasomotility, vasomotion, AVA and typical pathological EBDs (Stagnaro, 1985b, 2003, 2009d; Stagnaro-Neri and Stagnaro, 1989; Stagnaro and Caramel, 2012b).

In following, we briefly resume the easier way for the diagnosis of IRR of CVD: the Gastric Aspecific Reflex (G.A.R.) through the “Auscultatory Percussion of the Stomach” (Stagnaro, 2005).

In health, without any IRR of CVD, i.e., in absence of the “variant” Reaven's syndrome (Stagnaro, 2003), the intense digital pressure applied upon the projected skin area of any large artery (i.e., carotid artery) does not provoke a simultaneous gastric dilation and the contraction of the antral-pyloric region, i.e., negative biophysical semeiotic sign, absence of CVD and of ATS Constitution, termed negative Antognetti's sign (Stagnaro-Neri and Stagnaro, 1989; Stagnaro, 2009d; Stagnaro and Caramel, 2012b,e, 2013d), hence inducing local metabolic regulation of Tissue Microvascular Unit (T.M.U.), i.e., activating the Microcirculatory Functional Reserve (MFR) (Stagnaro, 1977; Stagnaro and Caramel, 2012b).

On the contrary, in subjects with ATS Constitution, under the above mentioned experimental condition, the identical maneuver brings about a simultaneous G.A.R.; i.e., the stomach dilates (less than 1 cm in case of IRR of CVD, at least 1 cm or more in case of overt ATS). Interestingly, such a reflex is smaller, less than 1 cm, if there is IRR of disease (Stagnaro and Caramel, 2012b), as in the case of Arrhythmic Constitution.

## Inherited real risk of cardiovascular disease: primary and pre-primary prevention

QBS tools are not only useful for diagnostic purposes, but also for therapeutic advices, because they are able to measure the microcirculatory activity before and after each preventive therapy's treatment, in order to understand the effectiveness of remedies.

One of the authors has recently discover a new class of treatments for preventive purposes termed “type B” or “blue” therapy (Stagnaro and Caramel, 2011c, 2013c,d,e), in accord with the Principle of Fractal Genome Recursive Function (PRGF) (Pellionisz, 2008), clinically corroborated by QBS experimental evidences (Stagnaro and Caramel, 2011d,e). The very initial, localized changes of artery wall, evaluated in “quantitative” way with a stethoscope during the first 10 years of life, playing a central role in atherogenesis, disappear, if recognized promptly, under the “blue” therapy. The “blue” therapy, used in a timely, optimal, and personalized manner, removes the IRR of CVD. Finally, QBS method makes possible and reliable the therapeutic monitoring of atherosclerotic lesions.
